# Validity and reliability of the movement behaviour questionnaire child in Chinese preschoolers

**DOI:** 10.3389/fped.2025.1544738

**Published:** 2025-03-21

**Authors:** Huiqi Song, Nike Lu, Jingjing Wang, Patrick W. C. Lau, Peng Zhou

**Affiliations:** ^1^ The Jockey Club School of Public Health and Primary Care, The Chinese University of Hong Kong, Hong Kong SAR, China; ^2^ Department of Sports and Health Sciences, Academy of Wellness and Human Development (Research), Faculty of Arts & Social Sciences, Hong Kong Baptist University, Hong Kong SAR, China; ^3^ Mass Sports Research Center, China Institute of Sport Science, Beijing, China; ^4^ Laboratory of Exercise Science and Health, Beijing Normal-Hong Kong Baptist University, Zhuhai, China

**Keywords:** reliability, validity, movement behaviour, preschool children, physical activity

## Abstract

**Background:**

The Movement Behaviour Questionnaire Child (MBQ-C) was developed to measure physical activity, screen time, and sleep in preschool children, but the Chinese version lacked validation.

**Methods:**

This cross-sectional study aimed to assess the validity and reliability of the open-ended version of the MBQ-C among Chinese preschoolers. Data were collected from 892 parents of children aged 0–5 years across 10 provinces via an online questionnaire. The MBQ-C includes items on physical activity, screen time, and sleep, and was validated against device-measured physical activity using accelerometers.

**Results:**

Internal consistency was high, with Cronbach's alpha values ranging from 0.80 to 0.88 for different sections. Test-retest reliability showed moderate intraclass correlation coefficients (ICCs) between 0.52 and 0.72. Confirmatory factor analysis indicated good construct validity (CFI = 0.95). Moderate significant correlations were found between MBQ-C reported physical activity and device-measured moderate-to-vigorous physical activity (*R* = 0.35, *p* < 0.001).

**Conclusion:**

The open-ended version of the MBQ-C demonstrates good validity and reliability in assessing movement behaviours among Chinese preschoolers. This tool is effective for proxy-reported measurements and can contribute to understanding and promoting healthy movement behaviours in early childhood.

## 1 Introduction

Physical inactivity, excessive recreational screen time, and insufficient sleep are key factors contributing to the increased prevalence of obesity and chronic disease risk among preschoolers (1–3). The World Health Organization (WHO) 24-h movement guidelines recommend that children under 5 years should engage in enough physical activity, get quality sleep, and limit excessive screen time (World Health Organization, 2019). However, measuring the 24-h movement behaviours of preschool children is challenging (4). The preferred measurement is the wearable device-based assessment, such as the accelerometers and pedometers, given their relatively objective and reliability in measuring physical activity and sedentary behaviour (5, 6). Nevertheless, this measurement cannot directly measure screen time exposures (7) and has limitations in detecting activities such as swimming or housework (8). In addition, the wearing position (waist, wrist, thighs) may cause the measured physical activity data to be biased (9, 10) and the overall quality of evidence on accelerometer cut-off points for measuring physical activity in preschool children is quite low (11). More importantly, accelerometers are difficult to apply to large populations due to their high-cost (12) and low compliance among preschool children (13). In contrast, proxy-report questionnaires may be a better option for measuring movement behaviours in preschoolers, as they are cost-effective and suitable for large population-based studies (14).

However, existing questionnaires for assessing preschoolers' physical activity, screen time, and sleep face several barriers. A systematic review assessed the quality of questionnaires on physical activity in children and found that the methodological quality of questionnaire studies in preschool children was poor, mainly due to inappropriate or unknown measurement properties of comparison measures and a lack of *a priori* hypotheses (15). Another systematic review on screen time measurement in young children found that most questionnaires primarily focus on the length of screen time, with few addressing frequency, content assessment, or co-viewing (16). Phillips et al. reviewed the instruments used to measure sleep in preschool children. They found that only a few studies have assessed the test-retest reliability and internal consistency of the questionnaires. Most studies assessed the convergent validity of the questionnaires by comparing them with accelerometers, but the validity of accelerometers for measuring sleep in this age group is unclear, making it difficult to accurately determine the validity of the questionnaires (17). Furthermore, there are very limited valid and/or reliable questionnaires to assess one or more of the 24-h movement behaviours of children aged under 5 years, and the quality of evidence was mostly low (18).

The Movement Behaviour Questionnaire Child (MBQ-C) is a pioneering validated proxy report developed in 2023 to rapidly assess 24-h movement behaviours in children aged 0–5 years (19). The MBQ-C is for toddlers and preschoolers who have reached movement development milestones (i.e., the ability to roll over, sit unsupported, crawl, stand unsupported, and walk unsupported). The questionnaire was designed through a cognitive interview phase involving parents to review and improve the content and clarity of the questionnaire items to ensure that the questionnaire was readable and understandable (19). In addition, specific examples related to the participants' daily lives, such as video calls, were included in the questionnaire to ensure the relevance and practicality of the content. The questionnaire also considers differences in behaviour between weekdays and weekends. This flexibility allows the questionnaire to capture children's movement patterns more comprehensively.

The MBQ-C has open-ended and closed-ended versions. Open-ended responses encourage parents to reflect on and remember their child's behaviour, while closed-ended responses offer specific choices to select from (19). The closed-ended version is more practical in clinical or primary care settings as it simplifies score calculation, thus assisting with goal setting and self-monitoring. The open version was primarily created for research purposes to collect statistical data that can be used to analyze differences within or between groups over time (7). The open-ended version was selected for validation primarily because it is designed for research applications where detailed statistical analysis is necessary. This version allows parents to detail their child's time spent on active play, screen time, and sleep over a typical day. Parents are prompted to provide their responses in hours and minutes, ensuring the data reflects actual time spent in these activities. The open-ended version of MBQ-C demonstrated good test-retest reliability, with an Intraclass Correlation Coefficient (ICC) ranging from 0.68 to 0.98 (7). Significant positive correlations were observed between screen time and sleep reported by the MBQ-C and the 24-h diary. Furthermore, there were positive correlations between MBQ-C and device measurements of total activity and energy playtime. The MBQ-C provides a feasible and cost-effective method for monitoring young children's health behaviours. To date, the Chinese version of the open-ended version of the MBQ-C has yet to be validated.

Therefore, this study aimed to examine the validity and reliability of the open-ended version of MBQ-C among Chinese preschoolers. The reliable and valid questionnaire will help us better understand movement behaviour and develop effective strategies to promote preschool children's health and well-being.

## 2 Methods

### 2.1 Study design

This study adopted a cross-sectional design. Ethical approval was obtained from the Research Ethics Committee of Hong Kong Baptist University. This study followed the Strengthening the Reporting of Observational Studies in Epidemiology (STROBE) statement.

### 2.2 Participants

This study was conducted in China's 10 municipalities or provinces (Beijing, Shanghai, Guangdong, Shandong, Jiangsu, Jiangxi, Henan, Hubei, Guizhou, and Hubei) and used snowball sampling. The online questionnaire was developed through the Wenjuanxing platform (https://www.wjx.cn/) and distributed via WeChat in the form of a QR code. The inclusion criteria for respondents were (1) being a parent of a child aged 0–5 years, (2) the child has reached his/her walking milestone, and (3) the child is typically a developing child without any physical or mental illness.

The power analysis was performed using an online sample size calculator (https://wnarifin.github.io/ssc/ssicc.html) (20). Given an expected reliability (ICC) of 0.5, a precision of 0.05, a confidence level of 95%, two repetitions per participants, and a dropout rate was 10%, 963 participants were required. A total of 962 parents viewed the online questionnaire. Of these, 892 consented electronically to participate in this study, 68 refused to participate, and 2 were excluded due to missing data. The final sample contained 892 participants with a response rate of 92.7%.

### 2.3 Measurements

#### 2.3.1 Open-ended version of MBQ-C

The open-ended version of MBQ-C is a parent-reported, 9-item assessment tool that consists of three domains: physical activity, screen time, and sleep. Specifically, 2 items are for active games, 2 for non-interactive screen time, 2 for interactive screen time, and 3 for sleep. The items for active play, non-interactive screen time, and interactive screen time are separate for weekdays and weekends. The three items for sleep are nighttime sleep, daytime sleep, and sleep patterns. Weighted averages of total active play, energetic play, passive screen time, sedentary passive screen time, interactive screen time, and sedentary interactive screen time were calculated. Total screen time is the sum of passive screen time and interactive screen time. Total sedentary screen time is the sum of sedentary passive screen time and sedentary interactive screen time. Total sleep time is the sum of nighttime sleep and daytime sleep. As suggested in the scoring guidelines, implausible or extreme values for weekday total active play, weekend day total active play, weekday energetic play, and weekend day energetic play are truncated.

#### 2.3.2 Device-measured physical activity

Physical activity was measured using the accelerometer (ActiGraph, GT3X-BT, Pensacola, FL, USA), which is a valid, cost-efficient, and widely used tool to assess physical activity level in preschoolers (21). The kindergarten teachers and parents received written and video instructions for the usage and placement of the accelerometer. Parents were asked to register an activity diary for both wear and non-wear time. Participants wore the accelerometer on the right hip to monitor all activities for seven consecutive days, except during periods of sleep and water-based activities. The ActiLife software (version 6.13) was used for device initialization, data reduction and data analysis.

A recording epoch of 15 s was used, and valid wear time was considered to be at least 8 h of wear time over at least three days (two weekdays and one weekend day). Non-wear time was defined by 20 consecutive minutes of zero count/minute. Accelerometers were initialized at a sampling rate of 30 Hz and then reintegrated into 60-s epochs for analysis (22). Time spent in different intensity domains was categorized using the cut-off points according to Butte et al.: sedentary: <819 counts per minute (CPM); light: 820–3,907 CPM; moderate: 3,908–6,111 CPM; and vigorous: ≥6,112 CPM (23). The sum of light intensity physical activity and moderate-to-vigorous physical activity was defined as total physical activity.

#### 2.3.3 Demographic information

Parent's age range, education level, child's date of birth, gender, height, weight, family structure, and the number of days their child attended childcare each week were collected through the online self-reported questionnaire. Besides, the respondent reported the family structure and number of children in the household aged 5 years or younger. The body mass index (BMI) was computed by dividing the body weight by the square of height (kg/m^2^).

### 2.4 Procedures

Data were collected between May 2024 and July 2024. First, the MBQ-C was translated and cross-culturally adapted. The MBQ-C was translated into Chinese by two professional translators who were familiar with the terminology and knew English but were native Chinese speakers. Cultural adaptations were made to the original question to adapt to the Chinese reality, such as replacing “FaceTime and Skype” with commonly used Chinese social applications such as “WeChat and QQ”. Then, the reconciled version of the instrument was back-translated to English by two independent professional translators. The translated questionnaire was pilot-tested with a convenience sample of 15 parents or parents to examine the comprehensibility, understandability, and equivalence of the instructions, items, and response options.

Then, the final version of the MBQ-C was assessed for its reliability and validity. After obtaining consent from the kindergarten, the researcher introduced and explained the study to the kindergarten teachers. Next, the kindergarten teachers sent the QR code of the online questionnaire to the WeChat group. Parents in the WeChat group filled out the questionnaire. Parents received written information about the purpose and procedures of the study before it began. In addition, parents were informed that their information was anonymous and that all responses would be kept confidential and used only for research purposes. Participants were allowed to withdraw from the study at any time. On the day the online questionnaire was distributed, 150 children were randomly selected to wear the accelerometer, and accelerometers were retrieved after seven days. A total of 250 parents were randomly selected for the reassessment of the MBQ-C during the seven-day recall period.

### 2.5 Statistical analysis

All data analyses were conducted using SPSS (version 29.0) and R language 4.3.3. Confidence interval of 95% was used, and *p* < 0.05 was considered statistically significant. Descriptive statistics were calculated for characteristics and variables. Continuous variables were presented with means and standard deviations, and categorical variables were described by counts and percentages.

A confirmatory factor analysis (CFA) was used to evaluate the construct validity. The criteria for the goodness of fit were comparative fit index (CFI, values >0.90), Tucker–Lewis Index (TLI, values >0.95), root mean square error of approximation (RMSEA, values <0.08), and non-normed fit index (NNFI, values >0.90) (24–26). The concurrent validity was determined by the associations between MBQ-C and the device-based physical activity measures using Spearman rank order correlation coefficients (correlation coefficients >0.40 representing good validity, 0.30–0.40 moderate validity, <0.30 poor validity) (27). Cronbach's alpha coefficient was used to evaluate internal consistency, with values greater than 0.70 considered acceptable (28). ICC was used for test-retest reliability analysis of the MBQ-C, with >0.75 presenting good reliability, 0.50–0.75 moderate reliability, and <0.5 poor reliability (29).

## 3 Results

### 3.1 Characteristics of the sample

The characteristics of the included parent-child dyads are presented in Table 1. Most parent respondents were female (82.8%) and aged between 26 and 35 (65.6%). More than half of the parent respondents had graduated from university. The proportion of small families (49.6%) and three-generation families (40.1%) was almost equal. The mean age of the children was 57.2 months and the child sample had a higher proportion of male children. The majority of children attended childcare 4–5 days per week. Most participating families (72.8%) had one child aged 5 years or younger.

**Table 1:** Demographic characteristics of the participants (*n* = 892).

Variables	Mean (SD)/Number (percentage)
Respondent role	
Mother	739 (82.8%)
Father	153 (17.2%)
Parent age (years)	
18–25	23 (2.6%)
26–35	585 (65.6%)
36–45	266 (29.8%)
>45	18 (2.0%)
Parent education level	
Secondary school or below	80 (9.0%)
Senior high school	216 (24.2%)
University undergraduate	536 (60.1%)
University postgraduate	60 (6.7%)
Family structure	
Single parent family	7 (0.8%)
Small family (living with parents)	442 (49.6%)
Intergenerational family (living with grandparents)	65 (7.3%)
Three-generation family (living with parents and grandparents)	358 (40.1%)
Others	20 (2.2%)
Child sex	
Male	470 (52.7%)
Female	422 (47.3%)
Child age (months)	57.2 (10.3)
Child weight (kg)	21.3 (7.8)
Child height (cm)	109.2 (10.8)
Child BMI (kg/m^2^)	18.1 (7.9)
Childcare attendance	
0 days	9 (1.0%)
1–3 days/week	14 (1.6%)
4–5 days/week	869 (97.4%)
Number of children in the household aged 5 years or younger	
1 child	649 (72.8%)
2 children	226 (25.3%)
≥3 children	17 (1.9%)

BMI, body mass index.

### 3.2 Descriptive statistics, floorand ceiling statistics

The descriptive statistics of each measurement are shown in Table 2. The mean time spent in active play was lower than the reference level according to the World Health Organization guidelines (147.8 min/day at baseline and 128.2 min/day at day 7) (30). The mean time spent in total sedentary screen time was higher than the recommended time (89.9 min/day at baseline and 72.7 min/day at day 7). Sleep time was more than 600 min per day. No evidence of floor or ceiling effects was identified in the physical activity domain in the MBQ-C (range: 0.1%–2.8%). The floor effect was found in the interactive screen time item (16.8% at baseline of participants) and non-interactive sedentary screen time (16.0% at day 7 of participants) of the screen time domain. The ceiling effect was found in the sleep routine item of the sleep domain (26.7% at baseline and 27.9% at day 7 of participants).

**Table 2:** Descriptive statistics, ceiling, and floor statistics.

Number	*N*	Mean (SD)	Median	25th percentile	75th percentile	Range	Floor, *n* (%)	Ceiling, *n* (%)
Physical activity								
Active play	872	147.8 (90.4)	124.0	81.4	180.0	471.4	0.2	0.1
Energetic play	868	73.8 (48.8)	60.0	38.6	90.0	280.0	2.1	0.1
Screen time								
Non-interactive	862	90.0 (69.8)	64.2	40.0	120.0	385.0	2.8	0.1
Interactive	869	53.4 (54.6)	38.6	10.0	66.0	293.7	16.8	0.1
Total screen	851	138.8 (103.3)	120.0	60.0	181.6	548.9	2.5	0.1
Sedentary screen time								
Non-interactive	874	58.4 (57.7)	42.9	17.1	77.1	315.7	0.3	0.1
Interactive	877	33.1 (44.7)	15.9	0.0	44.3	240.0	0.7	0.5
Total sedentary screen	872	89.8 (87.0)	62.0	25.0	120.0	495.0	8.1	0.1
Sleep								
Day sleep	889	91.5 (49.9)	112.0	60.0	120.0	244.0	0.1	0.1
Night sleep	845	523.5 (50.3)	540.0	480.0	544.0	573.0	1.2	0.1
Total sleep	845	614.2 (71.7)	601.0	570.0	660.0	482.0	0.1	0.1
Sleep routine	892	5.7 (1.9)	6.0	4.0	8.0	7.0	2.9	26.7
Day 7								
Physical activity								
Active play	211	128.2 (80.2)	116.1	61.9	139.9	371.7	0.9	0.5
Energetic play	211	68.6 (43.7)	60.0	31.4	77.3	222.9	2.8	0.5
Screen time								
Non-interactive	206	79.1 (50.9)	63.4	38.5	119.6	240.0	3.3	1.0
Interactive	206	61.3 (46.4)	60.0	22.1	90.3	208.4	9.5	0.5
Total screen	206	139.1 (85.4)	120.9	62.5	199.0	399.3	1.0	0.5
Sedentary screen time								
Non-interactive	206	44.1 (44.5)	32.1	9.0	68.8	180.0	16.0	0.5
Interactive	206	28.2 (29.5)	20.0	0.0	52.6	120.0	0.5	0.9
Total sedentary screen	206	72.7 (63.0)	60.0	27.2	103.1	265.7	0.5	0.5
Sleep								
Day sleep	199	103.3 (47.6)	120.0	90.0	120.0	240.0	0.5	1.0
Night sleep	199	529.9 (51.4)	540.0	480.0	553.5	300.0	2.6	0.5
Total sleep	199	629.4 (82.0)	640.0	600.0	671.3	610.0	0.5	0.5
Sleep routine	199	5.8 (1.8)	6.0	4.0	8.0	7.0	1.9	27.9

SD, standard deviation.

### 3.3 Internal consistency

The Cronbach's alpha was 0.81 for the physical activity section, 0.87 for the screen time section, 0.88 for the sleep section, and 0.80 for the MBQ-C. When deleted individually, the Cronbach's alpha values for each section and MBQ-C were calculated for each item. Deleting any item did not lead to an increase in the Cronbach's alpha for each section or MBQ-C.

### 3.4 Test-retest reliability

Seven days after the first questionnaire, 222 participants completed the open-ended version of MBQ-C again. The test-retest reliability results are shown in Table 3. ICCs for the open-ended version of the MBQ-C were moderate with ICCs ranging from 0.52 to 0.72.

**Table 3:** Test-retest reliability for the open-ended version of the MBQ-C.

Number	*N*	ICC (95% CI)
Physical activity		
Active play	211	0.66 (0.56, 0.74)
Energetic play	211	0.52 (0.36, 0.63)
Screen time		
Non-interactive	206	0.72 (0.63, 0.79)
Interactive	206	0.67 (0.57, 0.75)
Total screen	206	0.65 (0.53, 0.74)
Sedentary screen time		
Non-interactive	206	0.61 (0.49, 0.71)
Interactive	206	0.55 (0.41, 0.66)
Total sedentary screen	206	0.60 (0.48, 0.70)
Sleep		
Day sleep	199	0.72 (0.63, 0.79)
Night sleep	199	0.54 (0.39, 0.66)
Total sleep	199	0.64 (0.51, 0.73)
Sleep routine	199	0.62 (0.50, 0.71)

Deletions for missing data on Day 7 were as follows: physical activity (*N* = 11), non-interactive screen time (*N* = 16), interactive screen time (*N* = 16), sleep (*N* = 23).

### 3.5 Construct validity

**Figure 1:**
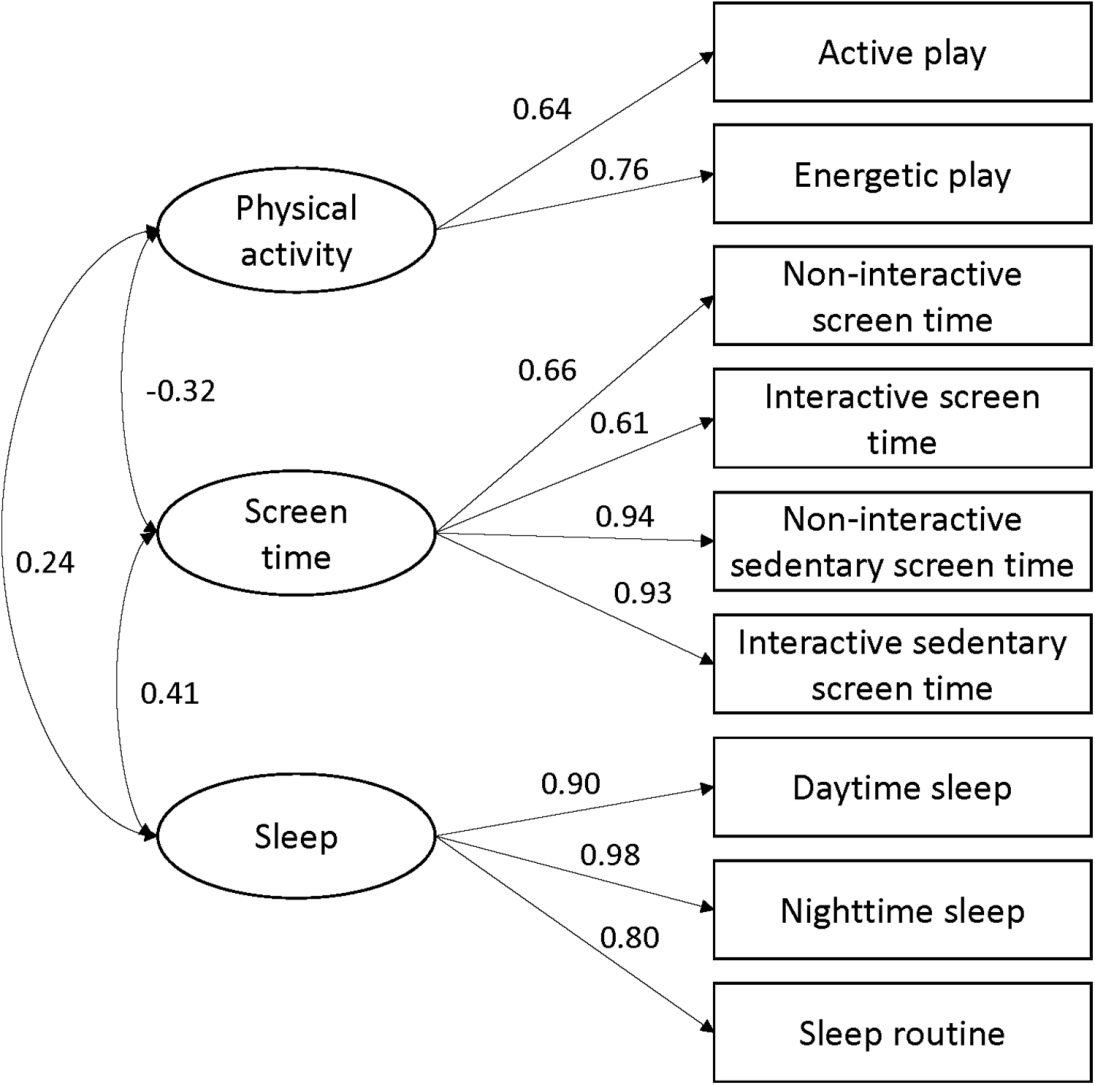
Confirmatory factor analysis of the open-ended version of movement behaviour questionnaire child (MBQ-C).

Confirmatory factor analysis showed the goodness of fit indexes for the scale were CFI = 0.95, TLI = 0.89, RMSEA = 0.11, and NNFI = 0.89. Factor loadings revealed significant relationships between the domains and items: physical activity (active play: 0.64, energetic play: 0.76), screen time (non-interactive screen time: 0.66, interactive screen time: 0.61, non-interactive sedentary screen time: 0.94, interactive sedentary screen time: 0.93), and sleep (daytime sleep: 0.90, nighttime sleep: 0.98, sleep routine: 0.80). Physical activity dimension was significantly correlated with screen time dimension (*r* = −0.32, *p* < 0.05), there was positive correlation between physical activity and sleep dimensions (*r* = 0.24, *p* < 0.05). Screen time dimension was positively correlated with sleep dimension (*r* = 0.41, *p* < 0.05) (see more details in Figure 1).

### 3.6 Concurrent validity

**Figure 2:**
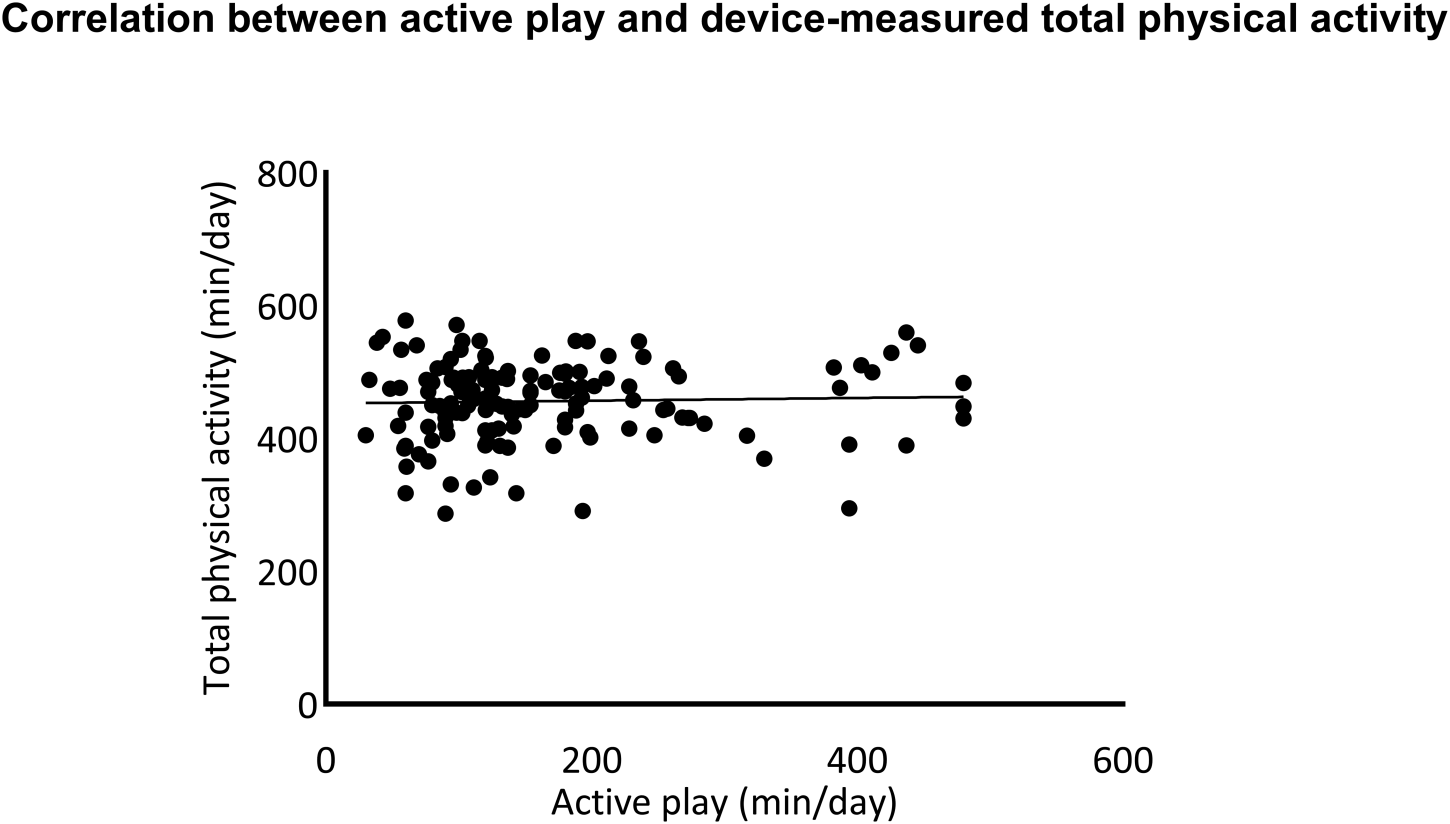
Correlation between the open-ended version of movement behaviour questionnaire child (MBQ-C) and device-measured total physical activity.

**Figure 3:**
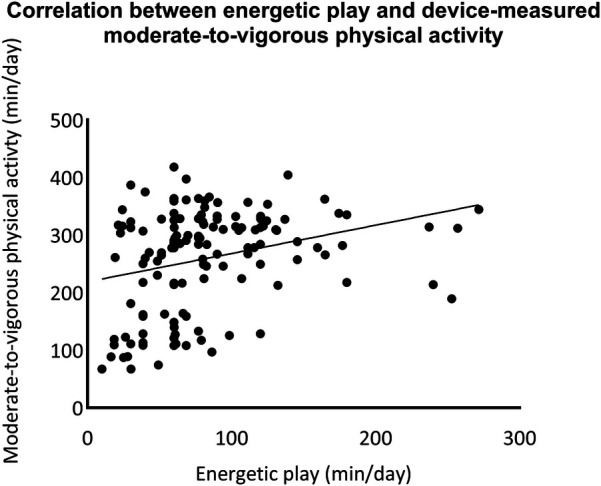
Correlation between the open-ended version of movement behaviour questionnaire child (MBQ-C) and device-measured moderate-to-vigorous physical activity.

A total of 139 children provided valid accelerometer data. The correlations between the open-ended version of the MBQ-C reported physical activity and device-measured physical activity were tested using Spearman's rank correlation. A moderate significant correlation was found in moderate-to-vigorous physical activity time (*R* = 0.35, *p* < 0.001). There was no significant correlation between parent-reported time in active play and device-measured total physical activity (see more details in Figures 2, 3).

## 4 Discussion

To the best of our knowledge, this is the first attempt to assess validity and reliability of the open-ended version of MBQ-C among Chinese preschoolers. The open-ended version of MBQ-C exhibited good internal consistency, moderate test-retest reliability, acceptable construct validity, and moderate concurrent validity for assessing physical activity, screen time, and sleep.

The internal consistency analyses indicated that all sections of the open-ended version of MBQ-C achieved acceptable Cronbach's alpha values (>0.80), which is consistent with recent studies measuring 24-h movement behaviours in young children using proxy-report questionnaires (16, 18). This result suggests that the items of the MBQ-C could effectively reflect the target constructs and remain stable.

In contrast to a previous study (7), this study reported moderate test-retest reliability for physical activity, screen time, and sleep section. Although ICC values for most items were higher than 0.60, some items (e.g., energetic play, interactive sedentary screen time, and night sleep) had low ICC values. The lower reliability observed in these outcomes might be partly due to floor effects and the limited variation in parent responses to these items. Since most children attended childcare regularly, parents completing the questionnaire may lack supervision and involvement in their children's daily activities. This finding suggests the need for caution in interpreting data when using questionnaire instruments, especially regarding dynamic and highly variable behaviours (7).

The open-ended version of MBQ-C was supported by construct validity, indicating the accuracy of measuring movement behaviors. This finding shows that this questionnaire appears to be a valid tool for assessing movement behaviors in the Chinese context. This finding also echoes the guidelines on children's movement behaviours, highlighting the importance of physical activity, adequate sleep, and limiting screen time in preschool children's healthy development (30).

This study reported a moderately significant association between the MBQ-C and device-measured physical activity, which is consistent with previous studies. Trost et al. found that the correlation between proxy-report physical activity and device-measured physical activity ranges from 0.25 to 0.39 (7). Another study reported a correlation of 0.39 between parent-reported physical activity and device-measured moderate-to-vigorous physical activity (31). This result indicates that both parent reports of preschool children's movement behaviours and device-measured data may be influenced by various biases, such as recall bias from parents and potential inaccuracies in device measurement (14). Different from sleep and screen time, it is difficult for parents to monitor their child's movement behaviours throughout the day, especially in the present study where more than 90% of the children attended kindergarten on a regular weekly basis. Besides, the episodic and pulsatile nature of physical activity in preschool children complicates parents' ability to accurately estimate its frequency, intensity, and duration (32). However, in cases where device-based measures of physical activity are not feasible, our findings suggest that the MBQ-C can be effective in assessing physical activity participation in children aged 5 years and younger.

This study is limited due to moderate sample size and convenience sampling, which may have introduced bias. In addition, although the study conducted data collection in multiple provinces and cities, there is still a need to validate the generalizability of the MBQ-C in a broader sample. The two-item physical activity dimension was derived from a prior instrument focused on frequency and duration of activity and captured the construct's essence while minimizing participant burden. However, the brevity of the physical activity dimension should be noted. Future studies can consider expanding the item pool for this construct to enhance the reliability and validity of the measurement. Considering the differences between physical activity measured by parent proxy questionnaires and devices, future studies should choose suitable measures based on specific study objectives and participant characteristics. Although combining these two methods may help provide more comprehensive and accurate data on preschoolers' movement behaviors, less biased measures are needed in the future to improve the reliability and validity of data.

## 5 Conclusion

In conclusion, the open-ended version of MBQ-C has good validity and reliability in Chinese preschoolers, and it is an effective proxy-reported measure of movement behaviours.

## Data Availability

The raw data supporting the conclusions of this article will be made available by the authors, without undue reservation.
